# Diagnostic value of routine blood tests in differentiating between SARS-CoV-2, influenza A, and RSV infections in hospitalized children: a retrospective study

**DOI:** 10.1186/s12887-024-04822-y

**Published:** 2024-05-13

**Authors:** Longli Huang, Cuiying Ye, Renxi Zhou, Zexuan Ji

**Affiliations:** Hangzhou Children’s Hospital, 201 Wenhui Rd, Hangzhou, Zhejiang China

**Keywords:** SARS-CoV-2, Influenza A, RSV, Hospitalized children, Blood parameters

## Abstract

**Background:**

The Severe Acute Respiratory Syndrome Coronavirus 2 (SARS-CoV-2), influenza A, and respiratory syncytial virus (RSV) infections have similar modes of transmission and clinical symptoms. There is a need to identify simple diagnostic indicators to distinguish these three infections, particularly for community hospitals and low- and middle-income countries that lack nucleic acid detection kits. This study used clinical data to assess the diagnostic value of routine blood tests in differentiating between SARS-CoV-2, influenza A, and RSV infections in children.

**Methods:**

A total of 1420 children treated at the Hangzhou Children’s Hospital between December 2022 and June 2023 were enrolled in this study, of whom 351 had SARS-CoV-2, 671 had influenza, and 398 had RSV. In addition, 243 healthy children were also collected. The blood test results of SARS-CoV-2 patients were compared to those of patients with influenza A and RSV and the healthy controls. The area under the receiver operating characteristic curve (AUC-ROC) was employed to evaluate each blood parameter’s diagnostic value.

**Results:**

Children with SARS-CoV-2 exhibited notably elevated levels of white blood cell (WBC) count, platelet (PLT) count, neutrophil count, and neutrophil-to-lymphocyte ratio (NLR) compared to influenza A patients (*P* < 0.05). In contrast, SARS-CoV-2 patients exhibited a decrease in the mean platelet volume to platelet count ratio (MPV/PLT) and the lymphocyte-to-monocyte ratio (LMR) when compared to other individuals (*P* < 0.05). These parameters had an AUC between 0.5 and 0.7. Compared to patients with RSV, SARS-CoV-2 patients had significantly higher MPV/PLT and significantly lower WBC, lymphocyte, PLT, LMR, and lymphocyte multiplied by platelet (LYM*PLT) values (*P* < 0.05). However, only LYM*PLT had an acceptable diagnostic value above 0.7 for all age groups. Compared to healthy children, children with COVID-19 exhibited elevated NLR and MPV/PLT levels, alongside decreased lymphocyte, PLT, LMR, and LYM*PLT values. (*P* < 0.05). The AUC of the LMR, LYM*PLT, and PLT were above 0.7 in all age groups, indicating promising diagnostic values.

**Conclusions:**

The routine blood parameters among patients with COVID-19, influenza A, and RSV differ significantly early in the disease and could be used by clinicians to discriminate between the 3 types of infection.

**Supplementary Information:**

The online version contains supplementary material available at 10.1186/s12887-024-04822-y.

## Introduction

The Severe Acute Respiratory Syndrome Coronavirus 2 (SARS-CoV-2) was the cause of the emergence of the coronavirus pandemic, also known as COVID-19. Although the COVID-19 pandemic is no longer considered a public health emergency as of 2022, the simultaneous outbreak of SARS-CoV-2 infection alongside the seasonal respiratory syncytial virus (RSV) and influenza viruses, has led to several hospitalization peaks in several pediatric wards globally [1]. The influenza viruses are categorized into three different types, and influenza A is most likely to cause regional outbreaks and epidemics [2]. On the other hand, almost every child gets infected with RSV prior to reaching the age of two, and thus, this infection poses a significant disease burden in young children [3]. However, since these three infections have similar symptoms, including cough, rhinorrhea, and fever, it is difficult to distinguish them based solely on clinical symptoms [4]. As a result, there is a need to identify these infectious diseases early to isolate the highly infectious cases and thus provide prompt treatment interventions.

Real-time reverse transcription-polymerase chain reaction (RT-PCR) is extensively recognized as the reference or the“gold-standard” for diagnosing these three viruses in clinical laboratories. This diagnostic test has demonstrated comparable or superior sensitivity compared to viral culture and can effectively detect the presence of the virus in both its active and dormant state [5–7]. However, in resource-limited areas and community hospitals, conducting these tests can pose challenges due to the limited availability of financial resources, personnel, and equipment. As a result, antigen-based diagnostics are often used to obtain rapid results in low-income community hospitals. However, although these tests have exhibited reasonable performance in confirming viral infections, they have a relatively low sensitivity and cannot be used to differentiate between the three infections [8–11]. Therefore, simple diagnostic indicators are needed to facilitate differential diagnosis, particularly in hospitals with limited resources.

Recent studies have shown that blood parameters obtained from routine blood tests hold significant predictive prognostic value in various diseases, including infection and cancer [12]. These tests are fast to perform and are easily available in primary hospitals. As a result, routine blood tests could provide a simple, cost-effective tool to obtain a differential diagnosis. Several studies have examined the importance of routine blood tests in diagnosing SARS-CoV-2 infections in adults [13–15]. However, to date, there is less research on children. Specific studies suggest that children who are infected with SARS-CoV-2, Influenza, and RSV have distinct blood parameter profiles and clinical characteristics. The research conducted by Hedberg P et al. [16] reveals that children infected with SARS-CoV-2 tend to be older and have a lower occurrence of chronic cardiac and respiratory problems compared to other viruses. Additionally, blood parameters could be used to differentiate between SARS-CoV-2 and other respiratory viruses. Leila Mohebi and her colleagues discovered that respiratory syncytial virus (RSV) infections in children might cause alterations in the parameters of a complete blood count, which may vary from the changes reported in infections caused by SARS-CoV-2 and Influenza [17]. The research published by Zhu R et al. showed that regular blood parameters can be used as an effective technique for quickly detecting influenza virus infection in children [2].

In this study, we sought to compare the routine blood parameters in hospitalized SARS-CoV-2, influenza A, and RSV-positive children to those in healthy children. Additionally, we also investigated the predictive value of the neutrophil-to-lymphocyte ratio (NLR), lymphocyte-to-monocyte ratio (LMR), mean platelet volume to platelet count ratio (MPV/PLT), lymphocyte count multiplied by the platelet count (LYM*PLT), platelet (PLT), white blood cell (WBC), and lymphocyte (LYM) serum levels for early differential diagnosis of influenza A, RSV and SARS-CoV-2 in children.

## Methods

### Selection criteria

This study included the participation of hospitalized children aged < 18 years who received treatment for SARS-CoV-2, influenza A, and RSV in Hangzhou Children’s Hospital from December 2022 to June 2023. The patient’s electronic medical records were examined to eliminate instances in which treatment regimens, such as antibiotics and antiviral treatment, were administered prior to blood collection to confirm that the patient had not undergone any prior treatment. Inclusion criteria were RT-PCR confirmation of COVID-19, influenza A, or RSV, and negative RT-PCR confirmation of other respiratory viral infections. Trauma and surgical patients, cancer patients undergoing chemotherapy, and those with systemic chronic systemic diseases including hypertension, anemia, diabetes, respiratory disease, and impaired renal function were excluded. Patients with bacteremia, sepsis, abscess, and other viral infections (e.g., Enterovirus 71(EV-71) and Adenovirus infection) were also excluded. In addition, healthy children who underwent a health checkup at the Hangzhou Children’s Hospital within the same period were enrolled as healthy controls (H-group) for comparison. All participants enrolled in the H-group had to have a negative RT-PCR for all viruses.

### Data collection

On admission, all patients had routine blood tests and respiratory specimens, including nasal and pharyngeal swabs. Blood detection was carried out using a routine analyzer (BC5100CRP, Mindray, China). The recorded parameters consisted of blood cell count, mean platelet volume (MPV), and hemoglobin levels (Hb). RT-PCR was performed on all swabs to test for SARS-CoV-2, influenza A, or RSV infection. Clinical data and demographic were obtained retrospectively from patients’ electrical medical records.

### Data analysis

The data were analyzed using the statistical package for social sciences software (IBM SPSS, Chicago, IL, USA) version 25 and the MedCalc® Statistical Software version 20.100. Since the routine blood parameters levels are age-dependent, we divided the children into four age groups: below 6 months, 6 to 24 months, 2 to 6 years, and 6 to 18 years. The normality of the continuous variables was examined. In the context of variables that follow a normal distribution, we used the mean ± standard deviation (SD) to provide a summary of the data. On the other hand, for non-normally distributed variable, we presented as the data as the median with interquartile ranges. Additionally, the categorical variables were represented in the form of percentages. The study conducted a comparison of the blood parameters among three types of infections and the control groups across four different age groups. The one-way analysis of variance (ANOVA) test was employed to assess the variations in blood parameters for variables that followed a normal distribution, while the Kruskal-Wallis test was used for variables that did not follow a normal distribution. The comparison of the categorical variables was performed using Pearson’s Chi-squared test. Subsequently, when significant differences emerged among the three infection groups and the H-group in one age group, we employed the Bonferroni test for multiple comparisons to discern which groups exhibited a statistically significant difference. A significance level of *P* < 0.05 was employed to determine the statistical significance.

The diagnostic value of each evaluated blood parameter was assessed by utilizing the area under the curve (AUC) of a receiver operator characteristic curve (ROC). The diagnostic utility of the blood parameters was categorized as follows: those with an AUC falling between 0.6 and 0.7 were considered acceptable, those within the range of 0.7 to 0.9 were deemed excellent, and those with an AUC above 0.9 were classified as outstanding. All experimental analyses yielded statistically significant results when the *p*-value was below 0.05. The study utilized DeLong’s test to assess the statistical significance of differences in the area under the curve (AUC) of blood parameters across the three infection groups and the healthy control group. The cut-off value was determined using Youden’s index.

## Results

### Patient characteristics

**Fig. 1:**
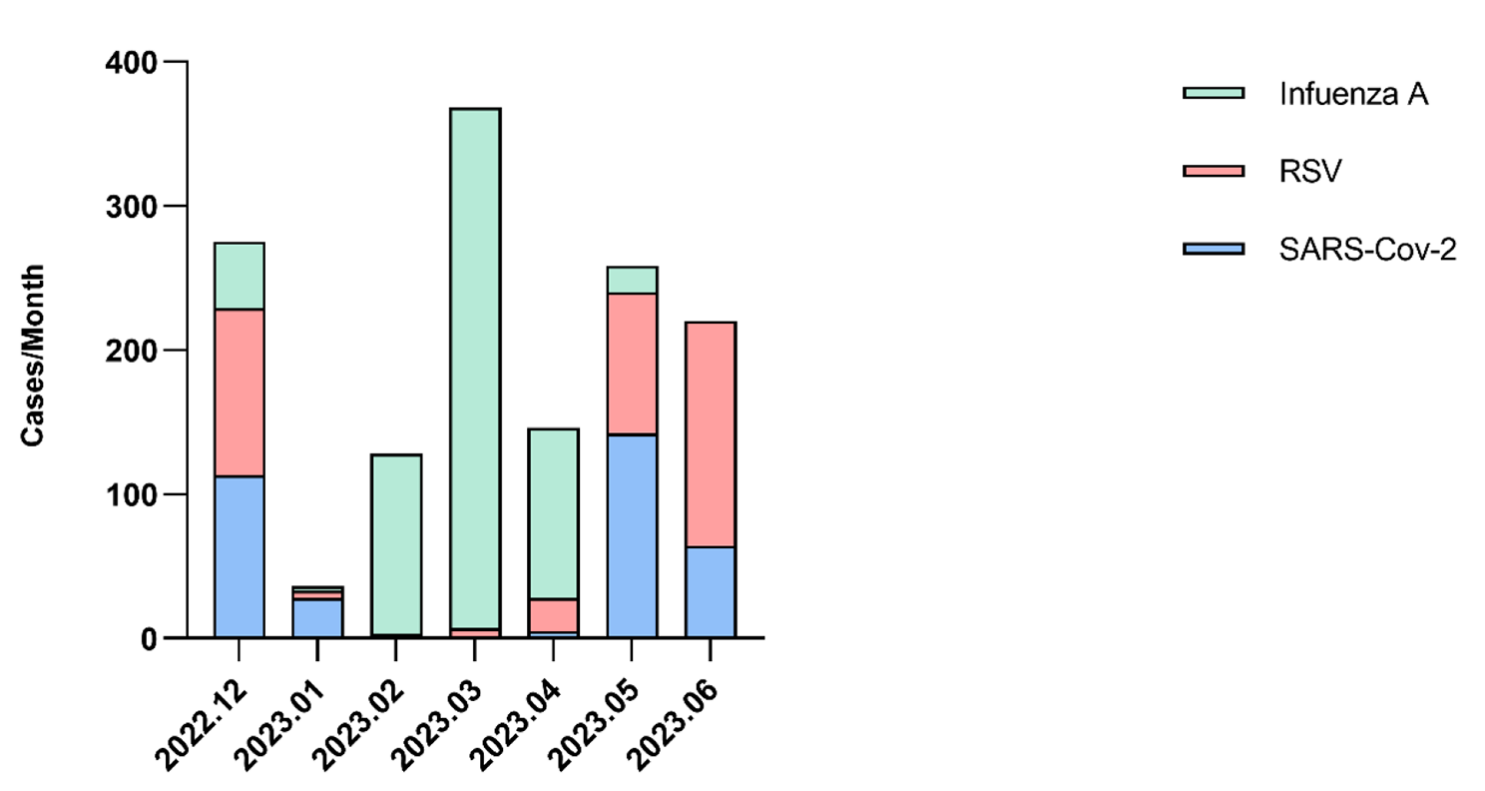
Monthly SARS-CoV-2, RSV, and influenza A cases in hospitalized children, December 2022 to June 2023

This cohort research comprised 1420 participants, among whom 351 individuals tested positive for SARS-Cov-2, 671 individuals tested positive for Influenza A, and 398 individuals were recognized as positive for RSV. (Fig. 1). Additionally, 243 children were enrolled in the healthy control group. There was no significant difference observed in gender distribution among the three infection groups and the H-group. However, a statistically significant difference in all age groups except the 6 to 24-month age group was noted between the three infection groups and the H-group (Table 1). We noted that children with SARS-CoV-2 were significantly younger (mean age, 11.94 ± 3.10 year) compared with influenza A (mean age, 4.51 ± 2.97 years) and healthy children (mean age, 3.33 ± 2.99 years) (Table 1).

### Monthly incidence of the SARS-CoV-2, RSV, and influenza A infections among the hospitalized children

Figure 1 demonstrates the number of children hospitalized per month for each pathogen. During the months of December 2022, May 2023, and June 2023, most hospitalizations were caused by the SARS-CoV-2 and RSV viruses. However, between February and March 2023, nearly all hospitalizations were caused by the influenza A virus.

**Table 1:** The baseline characteristics of the patients

		SARS-Cov-2 (*n* = 351)	Influenza A (*n* = 671)	RSV (*n* = 398)	H (*n* = 243)	X^2^/F	*P*-value
0 to 6 months (*n* = 235)	Female	74	6	22	6	6.245	0.100
	Male	80	16	29	2		
	Mean age(y)	0.20 ± 0.12^ac^	0.27 ± 0.10	0.23 ± 0.12	0.29 ± 0.08	4.408	0.005**
	no./total no. (%)	154/235(65.5)	22/235(9.4)	51/235(21.7)	8/235(3.4)		
6 to 24 months (*n* = 428)	Female	38	49	62	39	0.935	0.817
	Male	57	61	78	44		
	Mean age(y)	0.86 ± 0.22	0.87 ± 0.18	0.83 ± 0.19	0.82 ± 0.21	3.514	0.319
	no./total no. (%)	95/428(22.2)	110/428(225.7)	140/428(32.7)	83/428(19.4)		
2 to 6 years (*n* = 665)	Female	31	126	97	38	3.321	0.345
	Male	35	171	105	62		
	Mean age(y)	3.23 ± 1.13^b^	3.49 ± 1.06	2.70 ± 0.70	3.14 ± 0.98	69.035	0.000**
	no./total no. (%)	66/665(9.9)	297/665(44.7)	202/665(30.4)	100/665(15.0)		
6 to 18 years (*n* = 335)	Female	17	119	4	26	1.951	0.583
	Male	19	123	1	26		
	Mean age(y)	9.92 ± 2.89^abc^	8.00(6.00,9.00)	6.80 ± 1.10	8.19 ± 2.12	22.328	0.000**
	no./total no. (%)	36/335(10.7)	242/335(72.2)	5/335(1.5)	52/335(15.5)		
0 to 18 years (*n* = 1663)	Female	160	300	185	109	0.349	0.951
	Male	191	371	213	134		
	Mean age(y)	1.94 ± 3.10^ac^	4.51 ± 2.97	1.78 ± 1.28	3.33 ± 2.99	114.913	0.000**
	no./total no. (%)	351/1663(21.1)	671/1663(40.3)	398/1663(23.9)	243/1663(14.6)		

Abbreviations: SARS CoV-2: Severe acute respiratory syndrome coronavirus 2, RSV: respiratory syncytial virus, H health.

^a^Compared with the Influenza A group, *P* < 0.05

^b^Compared with the RSV group, *P* < 0.05

^c^Compared with the H group, *P* < 0.05

**P-value < 0.01.

### Comparison of routine blood parameters among the infected groups and H-group for the four age categories

The RBC counts in the SARS-CoV-2, influenza A, RSV, and H groups did not show a statistically significant difference in the below 6-month subgroup (Table 2). However, there were statistically significant differences in the RBC counts in the remaining three age subgroups (Tables 3 and 4, and 5). A statistically significant difference in the hemoglobin level (Hb) was only observed in the 6 to 24-month subgroup (Table 4). The remaining blood counts differed significantly between the four age groups (Tables 2, 3, 4 and 5). In the below 6-month subgroup, there were significant differences in the NLR and LMR between the SARS CoV-2 and influenza A infection groups. The WBC, LYM, PLT, LMR, and LYM*PLT parameters did not differ significantly between the SARS-CoV-2 and RSV groups. Conversely, a statistically significant difference was noted between the MPV/PLT and PLT parameters between the SARS-COV-2 and the H-group (Table 2, Supplemental Fig. 1). In the 6 to 24-month subgroup, a statistically significant difference was noted in the WBC, LYM, RBC, PLT, MPV/PLT, and LYM*PLT parameters between the SARS-CoV-2 and RSV groups. Similarly, a statistically significant difference was noted in the LYM, RBC, PLT, NLR, LMR, LYM*PLT, and MPV/PLT levels between the SARS-CoV-2 and the H-group (Table 3, Supplemental Fig. 1). In the 2 to 6-year subgroup, significant differences in the WBC, LMR, PLT, and MPV/PLT levels were observed between the SARS-CoV-2 and influenza A groups. Similarly, a statistically significant difference was noted in the PLT, LYM, LYM*PLT, LMR, and MPV/PLT between the SARS-CoV-2 and RSV groups. The PLT, LYM, LYM*PLT, LMR, and MPV/PLT levels also differed significantly between the SARS-CoV-2 and the healthy control groups (Table 4, Supplemental Fig. 1). In the above 6-year subgroup, a statistically significant difference was noted in the WBC, Neutrophil, PLT, NLR, and MPV/PLT levels between the SARS-CoV-2 and the influenza A groups, while a statistically significant difference was noted in the RBC count between the SARS-CoV-2 and the RSV groups. Moreover, a statistically significant difference was noted in the LMR, LYM*PLT, MPV/PLT, PLT, and LYM levels between the SARS-CoV-2 and H-group (Table 5, Supplemental Fig. 1).

**Table 2:** Hematological parameters in the infected groups and H-group in children below 6 months

Parameters	SARS-CoV-2 (*n* = 154)	Influenza A (*n* = 22)	RSV (*n* = 51)	H-Group (*n* = 8)	F/H	*p*-value
WBC (10^9^/L)	7.65 ± 2.79^b^	7.37 ± 2.95	9.14 ± 2.75	7.58 ± 1.02	15.159	0.002^**^
NEU (10^9^/L)	1.59 (0.94,1.62)	1.16 (0.63,1.79)	1.93 (1.48,2.54)	1.42 ± 0.52	13.464	0.004^**^
LYM (10^9^/L)	4.50 ± 2.45 ^b^	5.16 ± 2.22	5.59 ± 1.6	5.38 ± 0.99	13.804	0.003^**^
RBC (10^12^/L)	3.89 ± 0.58	4.08 ± 0.62	3.84 ± 0.4	4.33 ± 0.5	2.634	0.051
Hb (g/L)	114.83 ± 15.95	112.86 ± 12.96	112.43 ± 14.33	115.38 ± 9.23	1.094	0.779
PLT (10^9^/L)	306.66 ± 96.18^bc^	327.27 ± 87.68	438.77 ± 141.01	485.50 ± 122.37	45.720	0.000^**^
NLR	0.37 (0.18,1.05)^a^	0.18 (0.13,0.33)	0.35 (0.23,0.45)	0.27 ± 0.11	9.067	0.028^*^
LMR	5.37 (2.64,9.51) ^abc^	9.91 ± 5.79	8.13 ± 4.11	13.13 ± 3.87	22.926	0.000^**^
LYM*PLT	1408.8 ± 951.54 ^bc^	1807.15 ± 1098.43	2529.28 ± 1282.11	2585.89 ± 656.67	40.855	0.000^**^
MPV/PLT	0.031 (0.024,0.041) ^bc^	0.031 (0.024,0.035)	0.022 (0.016,0.028)	0.0118 ± 0.005	43.944	0.000^**^

Abbreviations: WBC: White blood cell count, NEU: Neutrophil count, LYM: Lymphocyte count, RBC: Red blood cell count, Hb: Hemoglobin, PLT: Platelet count, NLR: Neutrophil-to-lymphocyte ratio, LMR: Lymphocyte-to-monocyte ratio, LYM*PLT: Lymphocyte* platelet, MPV/PLT: Mean platelet volume/platelet ratio. F/H: ANOVA test F value or Kruskal-Wallis H value.

^a^Compared with the Influenza A group, *P* < 0.05

^b^Compared with the RSV group, *P* < 0.05

^c^Compared with the H group, *P* < 0.05

The absence of letter superscripts ^abc^ in routine blood parameter data indicates that there were no significant differences in post-hoc comparisons among the groups. For instance, the neutrophil count data in the table, without letter superscripts, indicate that the *p*-values for the comparisons between SARS-CoV-2 and Influenza A, SARS-CoV-2 and RSV, and SARS-CoV-2 and H-Group were all above 0.05. This suggests that there is no significant difference between the groups.

*P* < 0.05 *. P **<** 0.01 **

**Table 3:** Hematological parameters in infection groups and H-group in children aged between 6 to 24 months

Parameters	SARS-Cov-2 (*n* = 95)	Influenza A (*n* = 110)	RSV (*n* = 140)	H-Group (*n* = 83)	F/H	*p*-Value
WBC (10^9^/L)	7.43 ± 2.97 ^b^	7.18 ± 2.6	9.1 ± 2.93	8.06 ± 1.2	36.036	0.000^**^
NEU (10^9^/L)	2.45 ± 1.71	1.98 ± 1.5	2.57 ± 1.4	1.76 ± 0.62	22.312	0.000^**^
LYM (10^9^/L)	4.29 ± 2.28^bc^	4.45 ± 2.43	5.52 ± 2.19	5.43 ± 1.09	28.569	0.000^**^
RBC (10^12^/L)	4.48 ± 0.38 ^bc^	4.52 ± 0.34	4.64 ± 0.37	4.62 ± 0.31	5.320	0.001^**^
Hb (g/L)	116.98 ± 10.07	118.47 ± 9.3	118.27 ± 9.84	119.99 ± 8.99	6.126	0.106
PLT (10^9^/L)	261.96 ± 87.49 ^bc^	247.63 ± 80.08	339.87 ± 91.11	337.58 ± 78.03	95.016	0.000^**^
NLR	0.52 (0.25,0.86)^c^	0.32 (0.14,1.06)	0.41 (0.28,0.69)	0.35 ± 0.16	15.922	0.001^**^
LMR	8.31 ± 5.2 ^c^	9.52 ± 6.75	7.64 (5.30,10.49)	11.57 ± 3.82	32.768	0.000^**^
LYM*PLT	1199.91 ± 848.8 ^bc^	1177.63 ± 927.92	1935.77 ± 1046.08	1849.68 ± 629.7	73.001	0.000^**^
MPV/PLT	0.039 ± 0.016 ^bc^	0.041 ± 0.015	0.029 ± 0.009	0.028 ± 0.008	90.864	0.000^**^

Abbreviations: WBC: White blood cell count, NEU: Neutrophil count, LYM: Lymphocyte count, RBC: Red blood cell count, Hb: Hemoglobin, PLT: Platelet count, NLR: Neutrophil-to-lymphocyte ratio, LMR: Lymphocyte-to-monocyte ratio, LYM*PLT: Lymphocyte* platelet, MPV/PLT: Mean platelet volume/platelet ratio. F/H: ANOVA test F value or Kruskal-Wallis H value.

^b^Compared with the RSV group, *P* < 0.05

^c^Compared with the H group, *P* < 0.05

The absence of letter superscripts ^abc^ in routine blood parameter data indicates that there were no significant differences in post-hoc comparisons among the groups. For instance, the neutrophil count data in the table, without letter superscripts, indicate that the *p*-values for the comparisons between SARS-CoV-2 and Influenza A, SARS-CoV-2 and RSV, and SARS-CoV-2 and H-Group were all above 0.05. This suggests that there is no significant difference between the groups.

*P* < 0.05 *. P **<** 0.01 **

**Table 4:** Hematological parameters in infection groups and H-group in children aged between 2 to 6 years

Parameters	SARS-Cov-2 (*n* = 66)	Influenza A (*n* = 297)	RSV (*n* = 202)	H-Group (*n* = 100)	F/H	*p*-Value
WBC (10^9^/L)	6.82 ± 3.01^a^	5.41 ± 2.1	6.99 ± 2.31	6.81 ± 1.23	88.778	0.000^**^
NEU (10^9^/L)	3.03 ± 2.11	2.44 ± 1.82	3.05 ± 1.84	2.42 ± 0.84	23.140	0.000^**^
LYM (10^9^/L)	2.58 ± 1.31^bc^	2.37 ± 1.14	3.15 ± 1.22	3.72 ± 0.9	108.943	0.000^**^
RBC (10^12^/L)	4.48 ± 0.36	4.49 ± 0.31	4.47 ± 0.31	4.58 ± 0.29	10.803	0.013^*^
Hb (g/L)	122.49 ± 7.97	123.82 ± 7.93	121.75 ± 7.4	125.3 ± 7.46	16.708	0.001^**^
PLT (10^9^/L)	229.22 ± 57.49^abc^	205.98 ± 56.66	257.71 ± 58.96	301.51 ± 68.08	164.438	0.000^**^
NLR	1.04 (0.58,1.84)^c^	0.83 (0.35,1.98)	0.91 (0.52,1.47)	0.63 (0.47,0.85)	17.281	0.001^**^
LMR	5.00 ± 3.94 ^abc^	5.46 (2.95,8.34)	6.17 ± 3.34	8.83 ± 3.05	66.021	0.000^**^
LYM*PLT	602.07 ± 370.23^bc^	498.06 ± 304.63	830.84 ± 419.56	1130.76 ± 393.6	192.048	0.000^**^
MPV/PLT	0.041 (0.035,0.051) ^abc^	0.049 ± 0.016	0.038 ± 0.011	0.031 ± 0.010	154.539	0.000^**^

Abbreviations: WBC: White blood cell count, NEU: Neutrophil count, LYM: Lymphocyte count, RBC: Red blood cell count, Hb: Hemoglobin, PLT: Platelet count, NLR: Neutrophil-to-lymphocyte ratio, LMR: Lymphocyte-to-monocyte ratio, LYM*PLT: Lymphocyte* platelet, MPV/PLT: Mean platelet volume/platelet ratio. F/H: ANOVA test F value or Kruskal-Wallis H value.

^a^Compared with the Influenza A group, *P* < 0.05

^b^Compared with the RSV group, *P* < 0.05

^c^Compared with the H group, *P* < 0.05

The absence of letter superscripts ^abc^ in routine blood parameter data indicates that there were no significant differences in post-hoc comparisons among the groups. For instance, the neutrophil count data in the table, without letter superscripts, indicate that the *p*-values for the comparisons between SARS-CoV-2 and Influenza A, SARS-CoV-2 and RSV, and SARS-CoV-2 and H-Group were all above 0.05. This suggests that there is no significant difference between the groups.

*P* < 0.05 *. P **<** 0.01 **

**Table 5:** Hematological parameters in infection groups and H-group in children aged between 6 to 18 years

Parameters	SARS-Cov-2 (*n* = 36)	Influenza A (*n* = 242)	RSV (*n* = 5)	H-Group (*n* = 52)	F/H	*p*-Value
WBC (10^9^/L)	6.23 ± 2.54^a^	4.54 ± 1.91	7.65 ± 1.27	6.98 ± 1.32	77.101	0.000^**^
NEU (10^9^/L)	3.68 ± 2.36 ^a^	2.36 ± 1.91	3.62 ± 0.99	3.44 ± 1.09	45.048	0.000^**^
LYM (10^9^/L)	1.87 ± 1.07^c^	1.73 ± 0.83	3.22 ± 1.12	2.88 ± 0.7	67.298	0.000^**^
RBC (10^12^/L)	4.70 ± 0.45^b^	4.58 ± 0.35	4.27 ± 0.22	4.66 ± 0.38	2.957	0.033^*^
Hb (g/L)	131.18 ± 11.15	129.86 ± 9.05	122.6 ± 5.94	129.29 ± 10.28	1.275	0.283
PLT (10^9^/L)	231.03 ± 59.46^ac^	201.24 ± 57.49	285.4 ± 30.5	286.9 ± 50.64	81.622	0.000^**^
NLR	1.65 (1.03,3.37) ^a^	0.90 (0.52,2.29)	1.25 ± 0.61	1.09 (0.88,1.52)	11.088	0.011^**^
LMR	4.01 ± 2.25 ^c^	5.32 ± 3.42	5.94 ± 3.64	7.21 ± 2.31	32.415	0.000^**^
LYM*PLT	460.31 ± 315.52 ^c^	325.62 (182.79,474.16)	917.99 ± 326.24	835.38 ± 272.51	94.392	0.000^**^
MPV/PLT	0.045 ± 0.018^ac^	0.049 (0.040,0.061)	0.035 ± 0.006	0.032 (0.027,0.038)	75.164	0.000^**^

Abbreviations: WBC: White blood cell count, NEU: Neutrophil count, LYM: Lymphocyte count, RBC: Red blood cell count, Hb: Hemoglobin, PLT: Platelet count, NLR: Neutrophil-to-lymphocyte ratio, LMR: Lymphocyte-to-monocyte ratio, LYM*PLT: Lymphocyte* platelet, MPV/PLT: Mean platelet volume/platelet ratio. F/H: ANOVA test F value or Kruskal-Wallis H value.

^a^Compared with the Influenza A group, *P* < 0.05

^b^Compared with the RSV group, *P* < 0.05

^c^Compared with the H group, *P* < 0.05

The absence of letter superscripts ^abc^ in routine blood parameter data indicates that there were no significant differences in post-hoc comparisons among the groups. For instance, the neutrophil count data in the table, without letter superscripts, indicate that except for the SARS-CoV-2 and Influenza A group, the *p*-values for the comparisons between SARS-CoV-2 and RSV, and SARS-CoV-2 and H-Group were all above 0.05. This suggests that there is no significant difference between these groups.

*P* < 0.05 *. P **<** 0.01 **

### Diagnostic value of the routine blood parameters for distinguishing between the infected and healthy children

The AUC values of the different blood parameters for the four age groups are summarized in Tables 6, 7, 8 and 9.

For children below 6 months, the LMR, LYM*PLT, MPV/PLT, and PLT levels had an excellent diagnostic value (AUC above 0.8) to distinguish between the SARS-CoV-2 and H-group (Table 6). The LYM*PLT, MPV/PLT, and PLT levels had an adequate diagnostic value in distinguishing between SARS-CoV-2 and RSV, whereas the NLR and LMR values exhibited acceptable diagnostic value (AUC > 0.6) in differentiating between SARS-CoV-2 and influenza A.

**Table 6:** The highest AUROC between infection groups and H-group in children below 6 months of age

	AUC (95%CI)	Sensitivity (%)	Specificity (%)	Cut-off
**SARS-CoV-2 vs. H-Group**				
LMR	0.860(0.797–0.909)	69.5	100.0	8.58
LYM*PLT	0.860(0.796–0.909)	70.8	100.0	1742.3
MPV/PLT	0.911(0.856–0.950)	77.3	100.0	0.0235
Platelet count	0.881(0.821–0.927)	72.1	100.0	369*10^9^/L
**SARS-CoV-2 vs. Influenza A**				
NLR	0.662(0.587–0.731)	55.2	77.3	0.312
LMR	0.678(0.604–0.746)	44.8	86.4	4.52
**SARS-Cov-2 vs. RSV**				
LYM*PLT	0.767(0.703–0.823)	66.9	74.5	1642.2
MPV/*PLT	0.760(0.696–0.817)	51.9	88.2	0.0306
Platelet count	0.778(0.715–0.833)	57.8	86.3	315*10^9^/L

For the 6 to 24-month subgroup, the LYM*PLT, MPV/PLT, and PLT levels had an adequate diagnostic value (AUC 0.7 to 0.9) in distinguishing between the SARS-CoV-2, the healthy and RSV groups. However, the NLR and LMR parameters had a poor diagnostic value in distinguishing between SARS-CoV-2 and influenza A (AUC < 0.6) (Table 7).

**Table 7:** The highest AUROC between infection groups and H-group in children aged 6 to 24 months

	AUC (95%Cl)	Sensitivity (%)	Specificity (%)	Cut-off
**SARS-Cov-2 vs. H-Group**				
LMR	0.695(0.622–0.762)	43.2	97.6	6.09
LYM*PLT	0.744(0.673–0.806)	61.1	85.5	1246.3
MPV/PLT	0.752(0.681–0.813)	61.1	79.5	0.0325
Platelet count	0.749(0.679–0.811)	47.4	92.8	242*10^9^/L
**SARS-Cov-2 vs. Influenza A**				
NLR	0.564(0.493–0.633)	66.3	50.9	0.3245
Neutrophil count	0.582(0.511–0.650)	51.6	66.4	2.08*10^9^/L
**SARS-Cov-2 vs. RSV**				
LYM*PLT	0.715(0.652–0.772)	40.0	93.6	702
MPV/*PLT	0.720(0.658–0.777)	66.3	68.6	0.031
Platelet count	0.736(0.674–0.791)	63.2	76.4	279*10^9^/L

For children aged between 2 and 6 years, the LYM*PLT, MPV/PLT, LYM, and PLT levels had an excellent diagnostic value (AUC 0.7 to 0.9) to distinguish between the SARS-CoV-2, RVS, and the healthy controls groups (Table 8). The WBC, LMR, PLT, and MPV/PLT levels had an acceptable diagnostic value (AUC > 0.6) for distinguishing between SARS-CoV-2 and influenza A.

**Table 8:** The highest AUROC between infection groups and H-group in children aged 2 to 6 years

	AUC (95%Cl)	Sensitivity (%)	Specificity (%)	Cut-off
**SARS-Cov-2 vs. H-Group**				
LMR	0.842 (0.749–0.911)	77.8	82.7	4.9138
LYM*PLT	0.810 (0.712–0.886)	69.4	86.5	601.6
MPV/PLT	0.733 (0.628–0.821)	50.0	84.6	0.0406
Lymphocyte count	0.767 (0.665–0.850)	61.1	88.5	2.1*10^9^/L
Platelet count	0.752 (0.648–0.838)	66.7	71.2	258*10^9^/L
**SARS-Cov-2 vs. Influenza A**				
WBC count	0.697 (0.639–0.750)	77.8	63.6	4.61*10^9^/L
Platelet count	0.655 (0.596–0.711)	47.2	83.1	244*10^9^/L
MPV/PLT	0.662 (0.550–0.668)	69.4	68.2	0.0428
LMR	0.610 (0.550–0.668)	77.8	50.8	4.9138
**SARS-Cov-2 vs. RSV**				
LYM*PLT	0.839 (0.691–0.935)	63.9	100	490.68
Lymphocyte count	0.794 (0.640–0.904)	61.1	100	2.1*10^9^/L

For the 6 to 18 years age group, the LMR, LYM*PLT, and PLT levels had an excellent diagnostic value (AUC 0.7 to 0.9) for distinguishing between SARS-CoV-2 and the healthy control groups, while the RBC count had an excellent diagnostic value (AUC > 0.8) for distinguishing between the SARS-CoV-2 and RSV (Table 9). The WBC, PLT, and MPV/PLT levels had an acceptable diagnostic value (AUC > 0.6) for distinguishing between SARS-CoV-2 and influenza A.

**Table 9:** The highest AUROC between infection groups and H-group in children aged 6 to 18 years

	AUC (95%Cl)	Sensitivity (%)	Specificity (%)	Cut-off
**SARS-Cov-2 vs. H-Group**				
LMR	0.813 (0.746–0.870)	84.8	73.0	7.3286
LYM*PLT	0.844 (0.779–0.895)	66.7	90.0	630.72
Platelet count	0.793 (0.724–0.852)	77.3	76.0	260*10^9^/L
**SARS-Cov-2 vs. Influenza A**				
WBC count	0.639 (0.587–0.689)	69.7	51.2	5.13*10^9^/L
Neutrophil count	0.593 (0.540–0.644)	56.1	62.6	2.57*10^9^/L
Platelet count	0.625 (0.573–0.675)	63.6	59.9	209*10^9^/L
MPV/PLT	0.613 (0.561–0.663)	60.6	63.3	0.0429
**SARS-Cov-2 vs. RSV**				
RBC count	0.836 (0.687–0.933)	66.7	100.0	4.51*10^12^/L

## Discussion

Antigen and antibody detection and RT-PCR are the primary methods used to detect viral nucleic acid. However, not all hospitals have the equipment and personnel to perform RT-PCR, while antigen and antibody testing lack the sensitivity and specificity to distinguish between viral infections. Routine blood parameters could be used to distinguish between the different viruses. Although several studies have shown that routine blood parameters could be used to facilitate differential diagnosis in adults, the number of studies undertaken in the pediatric population remains limited [2, 14, 18].

Viruses are known to exhibit seasonal outbreak patterns. Prior to the advent of the COVID-19 pandemic, influenza epidemics in southern China usually occurred simultaneously in summer and winter, with a notable peak in summer [19]. On the other hand, RSV infections tended to occur between November and February [20]. In our study, we noted that following the COVID-19 outbreak, there has been a surge in the dissemination of the three viruses, particularly during the months of December 2022 and May 2023. However, the prevalence of the different infections varied between different age groups. Patients under 6 months of age were more likely to be hospitalized for SARS-CoV-2.

The assessment of blood parameters is of significant importance in the diagnosis of COVID-19 and the determination of patient prognosis. Although lymphopenia, leukopenia, neutropenia, and thrombocytopenia were reported in adults and children with COVID-19, these conditions tended to be less prevalent in children [21]. In our study, we compared the blood parameters in children who got infected with SARS-CoV-2 with those of healthy children from different age groups. Although no significant difference was found in the neutrophil count between the SARS-CoV-2 patients and the healthy children for all age groups, children older than 6 months had significantly lower LYM counts. Moreover, irrespective of the age group, significantly lower levels were noted for the MPV/PLT, LMR, and LYM*PLT between the SARS-CoV-2 patients and the healthy controls.

Although numerous studies have shown the strong predictive value of NLR [18], LMR [22], MPV/PLT [23], and LYM*PLT [23] parameters in the differential diagnosis of Covid-19 and forecasting the severity of COVID-19 in adults, the investigation of these parameters in children remains scarce. In our study, we assessed whether these blood parameters could be used to distinguish between children afflicted with SARS-Cov-2, RSV, and influenza A and healthy children. The LMR and LYM*PLT parameters can be readily derived from the whole blood count and are frequently employed as indicators of inflammation [24, 25]. Research has demonstrated that patients hospitalized for severe COVID-19 tend to suffer from lymphopenia and mononucleosis. These conditions tend to decrease the LMR and LYM count. Moreover, compared to healthy children, those with COVID-19 tended to have a significantly lower PLT count [21]. As a result, consistent with previous studies, the children with COVID-19 in our study tended to have a low LYM*PLT. Furthermore, the LYM*PLT levels also showed a good diagnostic value in distinguishing between SARS-CoV-2 and RSV-infected patients for all age groups. Research by Dejan Dobrijević et al. [26] indicates that platelet indices like mean platelet volume (MPV) and platelet distribution width (PDW) are independent predictors for COVID-19-positive children. This highlights the diagnostic significance of platelet indices in identifying and diagnosing COVID-19 in children. Our study further supported that MPV/PLT ratios exhibited notable distinctions between the SARS-CoV-2 and RSV groups within the 0–6 age bracket. Particularly, MPV/PLT levels were markedly higher in COVID-19-positive children compared to those with RSV, particularly in children under 2 years old, suggesting the potential of MPV/PLT as a tool for distinguishing between SARS-CoV-2 and RSV infections for diagnostic purposes. This disparity may be linked to increased secretion of proinflammatory cytokines and heightened platelet activation due to COVID-19 infection [26]. Our study findings indicate that the LMR, LYM*PLT, and PLT levels can differentiate between patients infected with SARS-CoV-2 and those who are healthy, regardless of age. However, these biomarkers are not effective in distinguishing between SARS-CoV-2 and influenza A infections.On the other hand, the RBC count showed excellent diagnostic value for distinguishing between SARS-CoV-2 and RSV infections in children above 6 years.

It is essential to recognize the presence of various limitations in this study. The limited sample size and the predominantly Zhejiang-based patient population included in this study can potentially restrict the applicability of our research findings to a broader context. We did not consider hospitalization caused by other infections common in children, such as rhinovirus and Mycoplasma pneumoniae. Therefore, further research with a larger sample and a more diverse patient cohort is recommended to enhance the generalizability of our research findings.

Research findings indicate the potential advantages of integrating Artificial Intelligence (AI) into the forecasting of COVID-19 consequences. AI’s ability to analyze data, recognize patterns, and process datasets may bolster the accuracy of risk evaluations. Amid the COVID-19 crisis, successful initiatives have employed automated machine learning to distinguish between influenza virus infections and SARS-CoV-2 [27], as well as promptly identifying COVID-19 in children [28, 29]. Advances have also been made in utilizing decision tree models based on hemogram outcomes to distinguish between RSV and COVID-19 in infants [30]. However, most of the above-mentioned AI model research was conducted in Europe, and its applicability to Asians is limited. We can use machine learning or other AI models to gain an in-depth understanding of the performance of the developed model on a global scale and understand its performance better. This technological integration can provide personalized risk predictions, guide targeted interventions, and revolutionize the management of SARS-CoV-2, Influenza, and RSV patients. This will also be one of the directions of our future research.

## Conclusion

COVID-19, influenza A, and RSV can cause various changes in peripheral blood parameters. Depending on the age group, several blood parameters, including LMR, LYM, LYM*PLT, MPV/PLT, WBC count, lymphocyte count, platelet count, and RBC count could be used to distinguish between COVID-19, influenza A, and RSV, with varying diagnostic efficacy. Our findings have the potential to assist clinicians in the early differentiation of these three diseases, thus facilitating prompt diagnosis and isolation. These blood parameters could also be used as a rapid screening tool in community hospitals and low- and middle-income countries where viral nucleic acid testing is limited.

### Electronic supplementary material

Below is the link to the electronic supplementary material.

Supplementary Material 1

## Data Availability

The datasets used and/or analyzed during the current study are available from the corresponding author upon reasonable request.

## Abbreviations

SARS-CoV-2: Severe Acute Respiratory Syndrome Coronavirus 2
RSV: respiratory syncytial virus
AUC: Area under the curve
WBC: White blood cell count
NEU: Neutrophil count
LYM: Lymphocyte count
RBC: Red blood cell count
Hb: Hemoglobin
PLT: Platelet count
NLR: Neutrophil-to-lymphocyte ratio
LMR: Lymphocyte-to-monocyte ratio
LYM*PLT: Lymphocyte* platelet
MPV/PLT: Mean platelet volume/platelet ratio
CI: Confidence interval
