# Supplementary material for: Diagnostic value of routine blood tests in differentiating between SARS-CoV-2, influenza A, and RSV infections in hospitalized children: a retrospective study
Source: BMC Pediatr. 2024 May 13;24:328. doi: 10.1186/s12887-024-04822-y (PMC11089714; doi:10.1186/s12887-024-04822-y)
Supplement: Supplementary file 1 — Supplementary Material 1 [file 12887_2024_4822_MOESM1_ESM.docx]

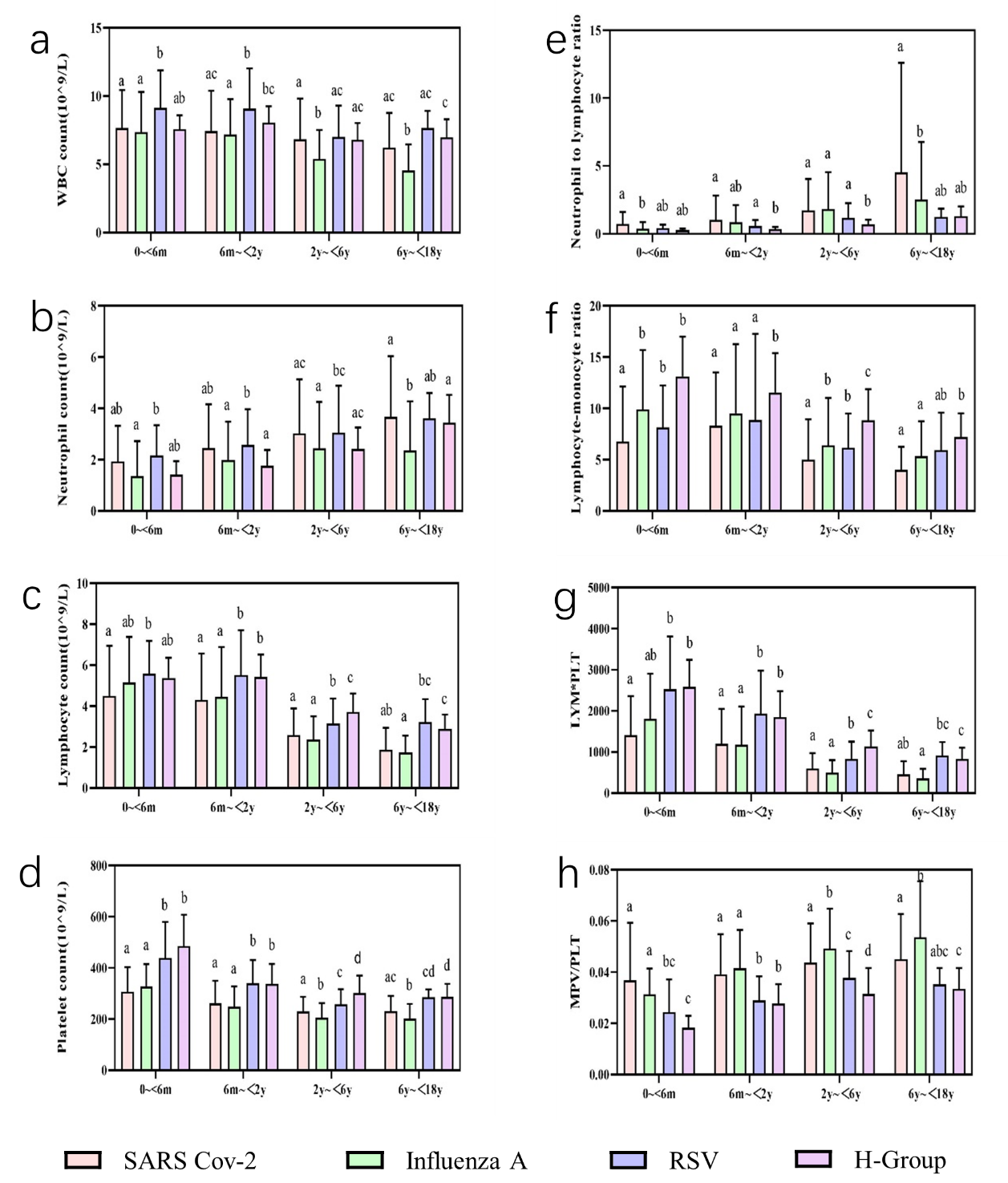


**Supplemental Fig 1.** Differences in the routine blood tests between the SARS-CoV-2, influenza A, RSV and healthy control groups

There are differences in the White Blood Cell (WBC) count(**Supplemental Fig 1a**), Neutrophil count (**Supplemental Fig 1b**), lymphocyte count (**Supplemental Fig 1c**), platelet count (**Supplemental Fig 1d**), neutrophil-to-lymphocyte ratio (NLR) (**Supplemental Fig 1e**), lymphocyte-to-monocyte ratio (**Supplemental Fig 1f**), lymphocyte*platelet (LYM*PLT) (**Supplemental Fig 1g**) and mean platelet volume-to-platelet ratio (MPV/PLT) values (**Supplemental Fig 1h**) between the SARS-CoV-2, influenza A, RSV and healthy control groups. We used different letters to denote significant differences in the histogram. Variables with the same letter indicate that the difference between the means is not statistically significant. Conversely, variables marked with different letters indicate that their mean values differed significantly.
